# Supplementary material for: Exploring the Accessory Genome of Multidrug-Resistant Rhodococcus equi Clone 2287
Source: Antibiotics (Basel). 2023 Nov 17;12(11):1631. doi: 10.3390/antibiotics12111631 (PMC10669575; doi:10.3390/antibiotics12111631)
Supplement: Supplementary file 1 [file antibiotics-12-01631-s001.zip › antibiotics-2724057-supplementary.pdf]

Supplementary Material

Table S1. Genomes of the 72 *R. equi* clinical isolates used in this study

Isolate	Sample Type	Year of Isolation	Genogroup	Macrolide-resistance mechanism	Accession no.	Reference
CL_mdr146	Clinical	2017	Clone 2287	*erm*(46)	SAMN13392179	[32]
CL_mdr147	Clinical	2017	Clone 2287	*erm*(46)	SAMN13392180	[32]
CL_mdr148	Clinical	2017	Clone 2287	*erm*(46)	SAMN13392181	[32]
CL_mdr152	Clinical	2017	Clone 2287	*erm*(46)	SAMN13392185	[32]
CL_mdr155	Clinical	2017	Clone G2016	*erm*(46)	SAMN13392188	[32]
CL_mdr156	Clinical	2017	Clone 2287	*erm*(46)	SAMN13392189	[32]
CL_mdr160	Clinical	2012	Clone 2287	*erm*(46)	SAMN13392193	[32]
CL_mdr161	Clinical	2013	Clone 2287	*erm*(46)	SAMN13392194	[32]
CL_mdr162	Clinical	2014	Clone 2287	*erm*(46)	SAMN13392195	[32]
CL_mdr163	Clinical	2014	Clone 2287	*erm*(46)	SAMN13392196	[32]
CL_mdr164	Clinical	2015	Clone 2287	*erm*(46)	SAMN13392197	[32]
CL_mdr165	Clinical	2017	Clone 2287	*erm*(46)	SAMN13392198	[32]
CL_mdr168	Clinical	2015	Clone 2287	*erm*(46)	SAMN13392201	[32]
CL_mdr169	Clinical	2015	Clone 2287	*erm*(46)	SAMN13392202	[32]
CL_mdr170	Clinical	2015	Clone 2287	*erm*(46)	SAMN13392203	[32]
CL_mdr171	Clinical	2015	Clone 2287	*erm*(46)	SAMN13392204	[32]
CL_mdr172	Clinical	2015	Clone 2287	*erm*(46)	SAMN13392205	[32]
CL_mdr173	Clinical	2015	Clone 2287	*erm*(46)	SAMN13392206	[32]
CL_mdr174	Clinical	2015	Clone 2287	*erm*(46)	SAMN13392207	[32]
CL_mdr176	Clinical	2017	Clone 2287	*erm*(46)	SAMN13392209	[32]
CL_mdr178	Clinical	2015	Clone 2287	*erm*(46)	SAMN13392211	[32]
CL_mdr180	Clinical	2015	Clone 2287	*erm*(46)	SAMN13392213	[32]
CL_mdr181	Clinical	2016	Clone 2287	*erm*(46)	SAMN13392214	[32]
CL_mdr183	Clinical	2016	Clone G2016	*erm*(46)	SAMN13392216	[32]
CL_mdr184	Clinical	2016	Clone 2287	*erm*(46)	SAMN13392217	[32]
CL_mdr185	Clinical	2016	Singleton	*erm*(46)	SAMN13392218	[32]
CL_mdr187	Clinical	2016	Singleton	*erm*(46)	SAMN13392220	[32]
CL_mdr188	Clinical	2017	Singleton	*erm*(46)	SAMN13392221	[32]
CL_mdr189	Clinical	2017	Singleton	*erm*(46)	SAMN13392222	[32]
CL_mdr191	Clinical	2017	Singleton	*erm*(46)	SAMN13392224	[32]
CL_mdr192	Clinical	2017	Singleton	*erm*(46)	SAMN13392225	[32]
CL_mdr194	Clinical	2017	Singleton	*erm*(46)	SAMN13392227	[32]
CL_s145	Clinical	2017	Singleton	Susceptible	SAMN13392178	[32]
CL_s149	Clinical	2017	Singleton	Susceptible	SAMN13392182	[32]
CL_s150	Clinical	2017	Singleton	Susceptible	SAMN13392183	[32]
CL_s151	Clinical	2017	Singleton	Susceptible	SAMN13392184	[32]
CL_s153	Clinical	2017	Singleton	Susceptible	SAMN13392186	[32]
CL_s154	Clinical	2017	Singleton	Susceptible	SAMN13392187	[32]
CL_s157	Clinical	2017	Singleton	Susceptible	SAMN13392190	[32]
CL_s158	Clinical	2017	Singleton	Susceptible	SAMN13392191	[32]
CL_s159	Clinical	2012	Singleton	Susceptible	SAMN13392192	[32]
CL_s166	Clinical	2015	Singleton	Susceptible	SAMN13392199	[32]
CL_s167	Clinical	2015	Singleton	Susceptible	SAMN13392200	[32]
CL_s175	Clinical	2015	Singleton	Susceptible	SAMN13392208	[32]
CL_s177	Clinical	2015	Singleton	Susceptible	SAMN13392210	[32]
CL_s179	Clinical	2015	Singleton	Susceptible	SAMN13392212	[32]
CL_s182	Clinical	2016	Singleton	Susceptible	SAMN13392215	[32]
CL_s186	Clinical	2016	Singleton	Susceptible	SAMN13392219	[32]
CL_s190	Clinical	2017	Singleton	Susceptible	SAMN13392223	[32]

CL_s193	Clinical	2017	Singleton	Susceptible	SAMN13392226	[32]
PAM 2282	Clinical	2011	Clone 2287	*erm*(46)	LWTT00000000	[35]
PAM 2289	Clinical	2010	Clone 2287	*erm*(46)	MUXK00000000	[35]
PAM 2291	Clinical	2010	Clone 2287	*erm*(46)	MVDS00000000	[35]
PAM 2292	Clinical	2010	Clone 2287	*erm*(46)	MVDT00000000	[35]
PAM 2293	Clinical	2011	Clone 2287	*erm*(46)	MVDU00000000	[35]
PAM 2294	Clinical	2011	Clone 2287	*erm*(46)	MVDV00000000	[35]
PAM 2295	Clinical	2011	Clone 2287	*erm*(46)	MVDQ00000000	[35]
PAM 2296	Clinical	2011	Clone 2287	*erm*(46)	MVDR00000000	[35]
PAM 2297	Clinical	2002	Clone 2287	*erm*(46)	MUXJ00000000	[35]
PAM2274	Clinical	2011	Singleton	Susceptible	LWTQ00000000	[35]
PAM2275	Clinical	2003	Clone 2287	*erm*(46)	MULU00000000	[35]
PAM2276	Clinical	2001	Singleton	Susceptible	LWTR00000000	[35]
PAM2277	Clinical	2004	Clone 2287	*erm*(46)	MUMB00000000	[35]
PAM2278	Clinical	2002	Singleton	Susceptible	MUMA0000000	[35]
PAM2279	Clinical	2001	Singleton	Susceptible	LWTS00000000	[35]
PAM2280	Clinical	2009	Clone 2287	*erm*(46)	MULW00000000	[35]
PAM2281	Clinical	2005	Clone 2287	*erm*(46)	MULT00000000	[35]
PAM2283	Clinical	2002	Clone 2287	*erm*(46)	MULY00000000	[35]
PAM2284	Clinical	2005	Clone 2287	*erm*(46)	MULZ00000000	[35]
PAM2285	Clinical	2005	Clone 2287	*erm*(46)	LWTU00000000	[35]
PAM2286	Clinical	2005	Clone 2287	*erm*(46)	MULX00000000	[35]
PAM2287	Clinical	2010	Clone 2287	*erm*(46)	LWTV00000000	[35]
ENV_mdr_1	Environmetal	2017	Clone 2287	*erm*(46)	WVDI00000000	[33]
ENV_mdr_8	Environmetal	2017	Clone 2287	*erm*(46)	WVDG00000000	[33]
ENV_mdr_9	Environmetal	2017	Clone 2287	*erm*(46)	WVEL00000000	[33]
ENV_mdr_10	Environmetal	2017	Clone 2287	*erm*(46)	WVDF00000000	[33]
ENV_mdr_12	Environmetal	2017	Clone 2287	*erm*(46)	WVDE00000000	[33]
ENV_mdr_14	Environmetal	2017	Clone 2287	*erm*(46)	WVDD00000000	[33]
ENV_mdr_16	Environmetal	2017	Clone 2287	*erm*(46)	WVDC00000000	[33]
ENV_mdr_18	Environmetal	2017	Clone 2287	*erm*(46)	WVDB00000000	[33]
ENV_mdr_20	Environmetal	2017	Clone 2287	*erm*(46)	WVEK00000000	[33]
ENV_mdr_22	Environmetal	2017	Clone 2287	*erm*(46)	WVDA00000000	[33]
ENV_s_23	Environmetal	2017	Clone 2287	*erm*(46)	WVAF00000000	[33]
ENV_mdr_24	Environmetal	2017	Clone 2287	*erm*(46)	WVCZ00000000	[33]
ENV_mdr_26	Environmetal	2017	Clone 2287	*erm*(46)	WVCY00000000	[33]
ENV_mdr_28	Environmetal	2017	Clone 2287	*erm*(46)	WVCX00000000	[33]
ENV_mdr_30	Environmetal	2017	Clone 2287	*erm*(46)	WVCW00000000	[33]
ENV_mdr_32	Environmetal	2017	Clone 2287	*erm*(46)	WVEJ00000000	[33]
ENV_mdr_36	Environmetal	2017	Clone 2287	*erm*(46)	WVCU00000000	[33]
ENV_mdr_40	Environmetal	2017	Clone 2287	*erm*(46)	WVCT00000000	[33]
ENV_mdr_55	Environmetal	2017	Clone 2287	*erm*(46)	WVCL00000000	[33]
ENV_mdr_58	Environmetal	2017	Clone 2287	*erm*(46)	WVCJ00000000	[33]
ENV_mdr_61	Environmetal	2017	Clone 2287	*erm*(46)	WVCH00000000	[33]
ENV_mdr_64	Environmetal	2017	Clone 2287	*erm*(46)	WVCF00000000	[33]
ENV_mdr_67	Environmetal	2017	Clone 2287	*erm*(46)	WVCD00000000	[33]
ENV_mdr_70	Environmetal	2017	Clone 2287	*erm*(46)	WVEG00000000	[33]
ENV_mdr_73	Environmetal	2017	Clone 2287	*erm*(46)	WVCA00000000	[33]
ENV_mdr_79	Environmetal	2017	Clone 2287	*erm*(46)	WVBX00000000	[33]

ENV_mdr_82	Environmetal	2017	Clone 2287	*erm*(46)	WVBV00000000	[33]
ENV_mdr_85	Environmetal	2017	Clone 2287	*erm*(46)	WVBT00000000	[33]
ENV_mdr_88	Environmetal	2017	Clone 2287	*erm*(46)	WVBR00000000	[33]
ENV_mdr_91	Environmetal	2017	Clone 2287	*erm*(46)	WVBP00000000	[33]
ENV_mdr_94	Environmetal	2017	Clone 2287	*erm*(46)	WVBN00000000	[33]
ENV_mdr_97	Environmetal	2017	Clone 2287	*erm*(46)	WVEE00000000	[33]
ENV_mdr_100	Environmetal	2017	Clone 2287	*erm*(46)	WVED00000000	[33]
ENV_mdr_106	Environmetal	2017	Clone 2287	*erm*(46)	WVBI00000000	[33]
ENV_mdr_109	Environmetal	2017	Clone 2287	*erm*(46)	WVBG00000000	[33]
ENV_mdr_112	Environmetal	2017	Clone 2287	*erm*(46)	WVBE00000000	[33]
ENV_mdr_115	Environmetal	2017	Clone 2287	*erm*(46)	WVBD00000000	[33]
ENV_mdr_118	Environmetal	2017	Clone 2287	*erm*(46)	WVBB00000000	[33]
ENV_mdr_124	Environmetal	2017	Clone 2287	*erm*(46)	WVAY00000000	[33]
ENV_mdr_127	Environmetal	2017	Clone 2287	*erm*(46)	WVAW00000000	[33]
ENV_mdr_130	Environmetal	2017	Clone 2287	*erm*(46)	WVAU00000000	[33]
ENV_mdr_133	Environmetal	2017	Clone 2287	*erm*(46)	WVAS00000000	[33]
ENV_mls_3	Environmetal	2017	Singleton	*erm*(46)	WVAM00000000	[33]
ENV_s_7	Environmetal	2017	Singleton	*erm*(46)	WVDW00000000	[33]
ENV_s_19	Environmetal	2017	Singleton	*erm*(46)	WVAH00000000	[33]
ENV_mdr_59	Environmetal	2017	Singleton	*erm*(46)	WVCI00000000	[33]
ENV_s_84	Environmetal	2017	Singleton	*erm*(46)	WUZL00000000	[33]
ENV_mdr_95	Environmetal	2017	Singleton	*erm*(46)	WVBM00000000	[33]
ENV_s_60	Environmetal	2017	Singleton	*erm*(46) & *erm*(51)	WVDS00000000	[33]
ENV_s_90	Environmetal	2017	Singleton	*erm*(46) & *erm*(51)	WUZJ00000000	[33]
ENV_s_105	Environmetal	2017	Singleton	*erm*(46) & *erm*(51)	WVDO00000000	[33]
ENV_mls_5	Environmetal	2017	Singleton	*erm*(51)	WVAL00000000	[33]
ENV_mdr_134	Environmetal	2017	Singleton	*erm*(51)	WVAR00000000	[33]
ENV_mdr_2	Environmetal	2017	Clone G2017	*erm*(51)	WVDJ00000000	[33]
ENV_mdr_6	Environmetal	2017	Clone G2017	*erm*(51)	WVDH00000000	[33]
ENV_s_15	Environmetal	2017	Clone G2017	*erm*(51)	WVAJ00000000	[33]
ENV_mdr_34	Environmetal	2017	Clone G2017	*erm*(51)	WVCV00000000	[33]
ENV_s_45	Environmetal	2017	Clone G2017	*erm*(51)	WUZV00000000	[33]
ENV_mdr_46	Environmetal	2017	Clone G2017	*erm*(51)	WVEH00000000	[33]
ENV_mdr_47	Environmetal	2017	Clone G2017	*erm*(51)	WVCQ00000000	[33]
ENV_mdr_49	Environmetal	2017	Clone G2017	*erm*(51)	WVCP00000000	[33]
ENV_mdr_50	Environmetal	2017	Clone G2017	*erm*(51)	WVCO00000000	[33]
ENV_s_51	Environmetal	2017	Clone G2017	*erm*(51)	WUZU00000000	[33]
ENV_mdr_53	Environmetal	2017	Clone G2017	*erm*(51)	WVCM00000000	[33]
ENV_mdr_56	Environmetal	2017	Clone G2017	*erm*(51)	WVCK00000000	[33]
ENV_mdr_62	Environmetal	2017	Clone G2017	*erm*(51)	WVCG00000000	[33]
ENV_mdr_65	Environmetal	2017	Clone G2017	*erm*(51)	WVCE00000000	[33]
ENV_mdr_68	Environmetal	2017	Clone G2017	*erm*(51)	WVCC00000000	[33]
ENV_mdr_71	Environmetal	2017	Clone G2017	*erm*(51)	WVCB00000000	[33]
ENV_mdr_74	Environmetal	2017	Clone G2017	*erm*(51)	WVEF00000000	[33]
ENV_mdr_77	Environmetal	2017	Clone G2017	*erm*(51)	WVBY00000000	[33]
ENV_mdr_80	Environmetal	2017	Clone G2017	*erm*(51)	WVBW00000000	[33]
ENV_mdr_83	Environmetal	2017	Clone G2017	*erm*(51)	WVBU00000000	[33]
ENV_mdr_86	Environmetal	2017	Clone G2017	*erm*(51)	WVBS00000000	[33]

ENV_mdr_89	Environmetal	2017	Clone G2017	*erm*(51)	WVBQ00000000	[33]
ENV_mdr_92	Environmetal	2017	Clone G2017	*erm*(51)	WVBO00000000	[33]
ENV_mdr_98	Environmetal	2017	Clone G2017	*erm*(51)	WVBL00000000	[33]
ENV_mdr_101	Environmetal	2017	Clone G2017	*erm*(51)	WVEC00000000	[33]
ENV_mdr_104	Environmetal	2017	Clone G2017	*erm*(51)	WVBJ00000000	[33]
ENV_mdr_107	Environmetal	2017	Clone G2017	*erm*(51)	WVBH00000000	[33]
ENV_mdr_110	Environmetal	2017	Clone G2017	*erm*(51)	WVBF00000000	[33]
ENV_mdr_113	Environmetal	2017	Clone G2017	*erm*(51)	WVEB00000000	[33]
ENV_mdr_116	Environmetal	2017	Clone G2017	*erm*(51)	WVBC00000000	[33]
ENV_mdr_119	Environmetal	2017	Clone G2017	*erm*(51)	WVEA00000000	[33]
ENV_mdr_122	Environmetal	2017	Clone G2017	*erm*(51)	WVAZ00000000	[33]
ENV_mdr_125	Environmetal	2017	Clone G2017	*erm*(51)	WVAX00000000	[33]
ENV_mdr_128	Environmetal	2017	Clone G2017	*erm*(51)	WVAV00000000	[33]
ENV_mdr_131	Environmetal	2017	Clone G2017	*erm*(51)	WVAT00000000	[33]
ENV_mdr_137	Environmetal	2017	Clone G2017	*erm*(51)	WVDZ00000000	[33]
ENV_mdr_140	Environmetal	2017	Clone G2017	*erm*(51)	WVAO00000000	[33]
ENV_mdr_142	Environmetal	2017	Clone G2017	*erm*(51)	WVAN00000000	[33]
ENV_s_4	Environmetal	2017	Singleton	Susceptible	WVAK00000000	[33]
ENV_s_13	Environmetal	2017	Singleton	Susceptible	WVDU00000000	[33]
ENV_s_25	Environmetal	2017	Singleton	Susceptible	WVAE00000000	[33]
ENV_s_27	Environmetal	2017	Singleton	Susceptible	WVAD00000000	[33]
ENV_s_29	Environmetal	2017	Singleton	Susceptible	WVAC00000000	[33]
ENV_s_31	Environmetal	2017	Singleton	Susceptible	WVAB00000000	[33]
ENV_s_33	Environmetal	2017	Singleton	Susceptible	WVAA00000000	[33]
ENV_s_39	Environmetal	2017	Singleton	Susceptible	WUZX00000000	[33]
ENV_s_42	Environmetal	2017	Singleton	Susceptible	WUZW00000000	[33]
ENV_s_48	Environmetal	2017	Singleton	Susceptible	WVDT00000000	[33]
ENV_s_57	Environmetal	2017	Singleton	Susceptible	WUZS00000000	[33]
ENV_s_63	Environmetal	2017	Singleton	Susceptible	WUZR00000000	[33]
ENV_s_65	Environmetal	2017	Singleton	Susceptible	WUZQ00000000	[33]
ENV_s_69	Environmetal	2017	Singleton	Susceptible	WUZP00000000	[33]
ENV_s_72	Environmetal	2017	Singleton	Susceptible	WUZO00000000	[33]
ENV_s_75	Environmetal	2017	Singleton	Susceptible	WVDR00000000	[33]
ENV_mdr_87_2	Environmetal	2017	Singleton	Susceptible	WUZK00000000	[33]
ENV_s_96	Environmetal	2017	Singleton	Susceptible	WUZI00000000	[33]
ENV_s_108	Environmetal	2017	Singleton	Susceptible	WUZG00000000	[33]
ENV_s_111	Environmetal	2017	Singleton	Susceptible	WVDN00000000	[33]
ENV_s_117	Environmetal	2017	Singleton	Susceptible	WUZE00000000	[33]
ENV_s_120	Environmetal	2017	Singleton	Susceptible	WUZD00000000	[33]
ENV_s_123	Environmetal	2017	Singleton	Susceptible	WVDM00000000	[33]
ENV_s_126	Environmetal	2017	Singleton	Susceptible	WUZC00000000	[33]
ENV_s_132	Environmetal	2017	Singleton	Susceptible	WUZB00000000	[33]
ENV_s_135	Environmetal	2017	Singleton	Susceptible	WUZA00000000	[33]
ENV_mdr_136	Environmetal	2017	Singleton	Susceptible	WVAQ00000000	[33]
ENV_s_138	Environmetal	2017	Singleton	Susceptible	WVDK00000000	[33]
ENV_s_141	Environmetal	2017	Singleton	Susceptible	WUYZ00000000	[33]
ENV_s_144	Environmetal	2017	Singleton	Susceptible	WUYY00000000	[33]
